# Excision of the Uvula and Tonsils

**Published:** 1876-02

**Authors:** 


Excision of the Uvula and Tonsils.—Mr. Sampson Gamagee, F.R.C.S., in an article upon the neglect of minor surgical operations, says: "The elongated uvula which troubles many orators and vocal artists, grows longer and more troublesome in spite of astringent lozenges and gargles. Its removal at just length, with a pair of scissors, is as nearly painless as can be possibly conceived, and is a lasting source of comfort. But of all conditions in which the superior economy of operative interference over miscalled palliative is demonstrable, none is so striking as chronic enlargement of the tonsils. Many children with pale faces and depressed chests, who speak thickly and snore in their sleep, have big, solid tonsils, which nearly touch in the middle line. It is beautiful to watch, in the course of a very few days, the cheeks becoming rosy and plump, and the chest expanding, after excision of the tonsils with the simple throat-guillotine, which in children and adults is one of the most useful yet insufficiently practiced operations. Doubtless the great question is when to interfere and when to abstain. That can only be solved by well-balanced judgment, based on ample information, neither stimulated by ambition for dexterous display nor checked by diffidence, which so frequently proclaims itself as the labored outcome of philosophical caution, when it is in reality the natural offspring of constitutional indecision.—*Louisville Medical News.*

